# Tailoring the Structure of Cell Penetrating DNA and RNA Binding Nucleopeptides

**DOI:** 10.3390/ijms23158504

**Published:** 2022-07-31

**Authors:** Stefano Tomassi, Caterina Ieranò, Alessandra Del Bene, Antonia D’Aniello, Maria Napolitano, Giuseppina Rea, Federica Auletta, Luigi Portella, Anna Capiluongo, Vincenzo Mazzarella, Rosita Russo, Angela Chambery, Stefania Scala, Salvatore Di Maro, Anna Messere

**Affiliations:** 1 Department of Pharmacy, University of Naples “Federico” II, Via D. Montesano 49, 80131 Napoli, Italy; stefano.tomassi@unina.it; 2 Molecular Immunology and Immunoregulation, Istituto Nazionale Tumori “Fondazione G. Pascale”, IRCCS-Napoli, 80131 Naples, Italy; c.ierano@istitutotumori.na.it (C.I.); m.napolitano@istitutotumori.na.it (M.N.); g.rea@istitutotumori.na.it (G.R.); f.auletta@istitutotumori.na.it (F.A.); l.portella@istitutotumori.na.it (L.P.); a.capiluongo@istitutotumori.na.it (A.C.); s.scala@istitutotumori.na.it (S.S.); 3 Department of Environmental, Biological and Pharmaceutical Sciences and Technologies, University of Campania “Luigi Vanvitelli”, Via Vivaldi 43, 81100 Caserta, Italy; alessandra.delbene@unicampania.it (A.D.B.); antonia.daniello@unicampania.it (A.D.); vincenzo.mazzarella@studenti.unicampania.it (V.M.); rosita.russo@unicampania.it (R.R.); angela.chambery@unicampania.it (A.C.)

**Keywords:** nucleic acid binders, nucleopeptides, cell penetrating peptide synthesis, cell penetrating peptides, nuclear localization sequences

## Abstract

Synthetic nucleic acid interactors represent an exciting research field due to their biotechnological and potential therapeutic applications. The translation of these molecules into drugs is a long and difficult process that justifies the continuous research of new chemotypes endowed with favorable binding, pharmacokinetic and pharmacodynamic properties. In this scenario, we describe the synthesis of two sets of homo-thymine nucleopeptides, in which nucleobases are inserted in a peptide structure, to investigate the role of the underivatized amino acid residue and the distance of the nucleobase from the peptide backbone on the nucleic acid recognition process. It is worth noting that the CD spectroscopy investigation showed that two of the reported nucleopeptides, consisting of alternation of thymine functionalized *L*-Orn and *L*-Dab and *L*-Arg as underivatized amino acids, were able to efficiently bind DNA and RNA targets and cross both cell and nuclear membranes.

## 1. Introduction

The essential role of RNA and DNA in many physio-pathologic conditions justifies the intensive studies aimed to disclose the molecular basis of the interactions between nucleic acids and their modulators [1,2]. Nowadays, the modulation of specific genes is considered a well-established strategy to treat diseases, including cancer, the onset and progression of which result from aberrant genetic regulation [3,4]. In this respect, the understanding that nucleic acids are modulated through the interaction with other nucleic acids and proteins paved the way for the development of synthetic nucleic acid binders. Among them, synthetic oligonucleotides and their analogues, including Locked Nucleic Acid (LNA), Morpholino Phosphorodiamidate Oligomers (MPOs) and Peptide Nucleic Acid (PNA), guarantee sequence specific recognition by base paring interactions and formation of stable complexes with nucleic acid regions [5,6]. Alternatively, nucleic acid-binding peptides, including cationic peptides, can bind DNA and RNA by electrostatic, hydrophobic, and hydrogen bond interactions, forming high stable complexes [7]. However, both oligonucleotide analogues and nucleic acid-binding peptides are affected by significant pharmacokinetic and pharmacodynamic limits, which often prevent their translation into a preclinical and clinical context. In particular, the first exhibit poor cell permeability and trafficking, while the latter are deficient in terms of biological stability and selectivity toward nucleic acids [8]. Taking into account that efficient cellular delivery, high binding affinity and selectivity are fundamental prerequisite of nucleic acid-targeted therapeutics, many efforts have been devoted to developing strategies, including chemical modification [6,9,10], conjugation and non-covalent complexation with carrier peptides and lipid transfection, that have partially addressed the aforementioned issues [11].

An intriguing compromise between oligonucleotides and peptides could be represented by a class of synthetic oligomers known as nucleopeptides in which nucleobases are inserted into a peptide structure [12,13]. Nucleopeptides were first introduced in the 1970s [12,13,14,15] but only recently have they been reconsidered as valuable alternative nucleic acid binders [16,17]. Electrostatic, hydrophobic, π-stacking and hydrogen-bond interactions are combined in nucleopeptide molecules, preserving both the cell penetrating peptide (CPP) features critical for cell uptake and the ability to recognize nucleic acids. In such a structure, the nucleobase-bearing amino acids (NBA) are included as additional recognition elements at defined positions within a peptide backbone. Although, several nucleopeptides do not form a canonical Watson–Crick (WC)-type regular structure when hybridizing with nucleic acids, they still take advantage of the presence of a nucleobase that can assure base pairing, and of the flexibility of the peptide backbone, which enables the conformational changes during the interaction with complementary single-strand DNA/RNA sequences. As recently described by us and others, nucleopeptides can be considered potential agents to modulate maturation processes of eukaryotic mRNAs, long non-coding RNA and microRNAs (miRNAs), because they are able to preferentially bind RNA, cross cell membrane and localize into the nucleus, when cationic amino acids are selected as underivatized residues [16,17]. Previous studies [18] proposed the nucleopeptides as new carriers for those nucleic acid analogues, especially PNA, whose delivery in the cell is still challenging. Further, stereochemical investigations indicated the key role of the backbone stereochemistry in the formation of the *L*-nucleopeptide/RNA complexes [19]. Finally, nucleopeptides can form supramolecular hydrogels representing an effective approach to creating new biomaterials [20,21] and to discovering novel anticancer agents based on ATP sequestration and self-assembling capability [22]. Unfortunately, the development of nucleopeptides goes unfilled probably as a consequence of the unclarified role of positive charged amino side chains in the selectivity of the recognition process of the target, in cell permeability and eventually in non-specific cytotoxicity. Moreover, few studies involving modified PNA oligomers showed that nucleobase-linker length affected the pairing selectivity of modified PNAs by altering the donor/acceptor positions at the Watson–Crick site as well as the orientation of Hoogsteen sites [23,24]. These results offer a valuable platform upon which to investigate the influence of the linker length between backbone and nucleobase on pairing selectivity and base pair stacking in the nucleopeptide oligomers. In our opinion, notable obstacles on the way to unveil nucleopeptide potentialities could be ascribed to the lack of examples of efficient synthetic methods. In this respect, we have already reported a powerful solid phase strategy [17] for the synthesis of nucleopeptides that enables these modifications, including the replacement of the underivatized amino acid residues and the modification of the number of the separating bonds between nucleobases and backbone. Thus, to identify a novel DNA and RNA-binder nucleopeptide featuring unprecedented structural characteristics, a 12-mer homo-thymine nucleopeptide model was considered for a two-phase optimization study involving: (i) the role of the underivatized amino acid residue in the binding process; and (ii) the distance of the nucleobases from the peptide backbone. Two sets of compounds were prepared and screened for their ability to recognize DNA and RNA. The best binders were selected to test their ability to cross the cellular and nuclear membranes.

## 2. Results and Discussion

### 2.1. Design and Synthesis

To optimize the ratio between the nucleic acid recognition and cell permeability of nucleopeptides, we designed and synthesized two small libraries of congeners. In the first, the L-Lys was conserved as thymine-functionalized amino acid, while the L-Arg residue was replaced with amino acids bearing charged polar side chains, including anionic (L-Asp and L-Glu) and cationic (L-Lys and L-His). The selection of these amino acids is dictated by two main considerations: (i) cationic and anionic amino acids are components of cell penetrating peptides [25]; (ii) in our previous study, the introduction of nonpolar amino acids, such as L-Ala, as well as polar amino acids, such as L-Ser and L-Gln [17], provided nucleopeptides affected by insufficient solubility in an aqueous medium or poor cellular uptake.

The second set of molecules was obtained by keeping L-Arg as underivatized cationic residue and replacing the L-Lys as thymine-functionalized amino acid with its lower homologues, such as the l-2,3-Diaminopropionic acid (L-Dap), the l-2,4-Diaminobutyric acid (L-Dab) and the L-Ornitine (L-Orn). This strategy enabled us to systematically modify the distance of the nucleobase from the backbone by progressively dropping the number of methylene groups of the side chain. With this approach, the amide linkage with the thymine-1-acetic acid was conserved in order to strictly evaluate the sole effects due to the different spacer lengths.

**Scheme 1:**
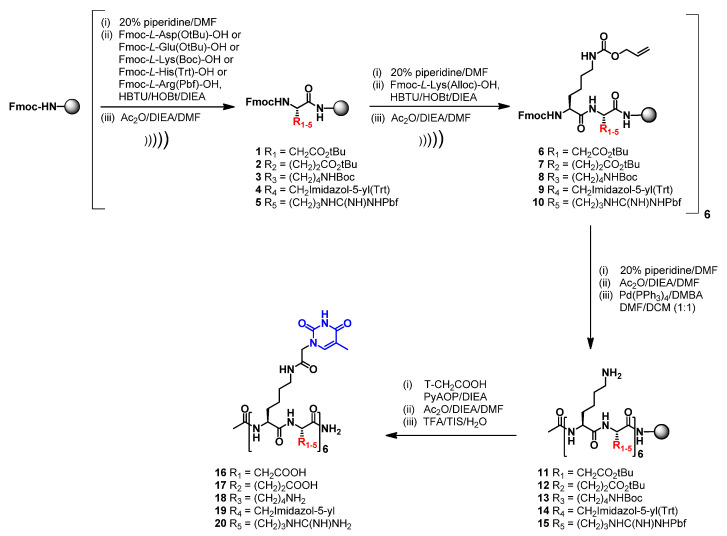
US-SPPS of nucleopeptides **16**–**20**.

The synthesis of both nucleopeptide libraries was fully accomplished by a solid-phase approach, improving our previously reported procedures [17,20,21]. In particular, a conventional synthetic approach for peptide synthesis was replaced by the US-SPPS strategy [26]. As described in Scheme 1, the 12-mer amino acid backbone of the nucleopeptides consisting of the alternation of nucleobase functionalized L-Lys and L-Asp (**16**) or L-Glu (**17**) or L-Lys (**18**) or L-His (**19**) or L-Arg (**20**) as underivatized amino acids was assembled through US-SPPS synthesis using a Rink-amide-aminomethyl polystyrene (AM-PS) resin, *N*,*N*,*N*′,*N*′-tetramethyl-*O*-(1H-benzotriazol-1-yl)uronium hexafluorophosphate (HBTU)/1-hydroxybenzotriazole (HOBt), and *N*,*N*-Diisopropylethylamine (DIEA) as activating/additive system. Briefly, the peptides were synthesized by using reaction times of 10 min in the presence of an excess of reagents (3 equiv. of Fmoc-aa and 3 equiv. HBTU/HOBt; 6 equiv. of DIEA). Complete Fmoc-removal was accomplished by using 20% pip/DMF solution, in 30 + 60 *s* by ultrasonic irradiation. After acetylation of the N-terminus, all the amino side chains were simultaneously deprotected using a DMF/DCM solution of Pd(PPh_3_)_4_ in the presence of *N*,*N*-Dimethylbarbituric acid (DMBA) as a scavenger. This deprotection was repeated two times for 60 min to assure the complete removal of Alloc protecting groups as confirmed by HPLC-MS analyses. Next, the thymine-1-acetic acids (T-CH_2_COOH) were introduced by one-pot reaction using PyAOP (3 equiv. for each amine group) and DIEA (6 equiv. for each amine group) for 8 h.

**Scheme 2:**
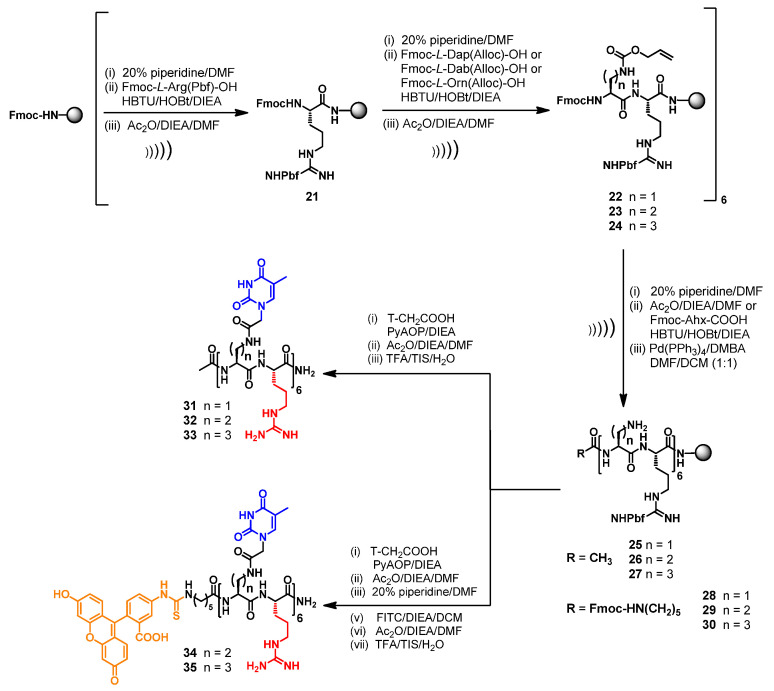
US-SPPS of nucleopeptides **31**–**35**.

The same strategy was successfully exploited for the synthesis of nucleopeptides consisting of the alternation of L-Arg and L-Dap (**31**) or L-Dab (**32**) or L-Orn (**33**) (Scheme 2). Finally, we also synthesized FITC- labelled derivatives (**34** and **35**) which were designed by selecting the structural features of the best DNA and RNA binders (**32** and **33**, respectively) that emerged during the first round of CD spectroscopic studies.

All the nucleopeptides were eventually removed from the solid support, purified by RP-HPLC, characterized by MALDI-MS and exhibited suitable solubility in water for CD (**16**–**20** and **31**–**33**) and cell-based studies (**34**–**35**).

### 2.2. Conformational Studies and Nucleic Acid Binding Properties of Nucleopeptides

**Figure 1:**
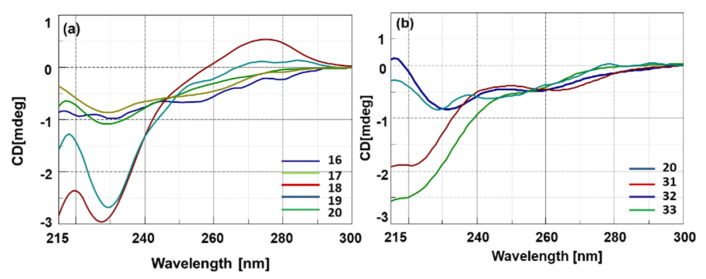
CD spectra of **16**–**20** (**a**) and **20**, **31**–**33** (**b**) nucleopeptides.

All the CD analyses were carried out using 10 μM concentration of nucleopeptides in 10 mM phosphate buffer (pH = 7.0) at 20 °C. As expected, CD spectra of single stranded nucleopeptides **16**–**20** and **31**–**33** indicated the absence of any preferential structural pre-organization (Figure 1), with only **18** and **19** that showed a more intense minimum at 228 nm (Figure 1a). Interestingly, the nucleopeptides **20** and **31**–**33** exhibited singular CD profiles depending on the number of methylene groups in the NBA side chains. Indeed, nucleopeptides formed by L-Dap and L-Orn (**31** and **33**, containing one and three methylene groups, respectively) exhibited a negative band in the 215–230 nm region, while *L*-Dab and *L*-Lys containing nucleopeptides (**32** and **20**, having two and four methylene groups, respectively) displayed a positive band in the same region (Figure 1b).

**Figure 2:**
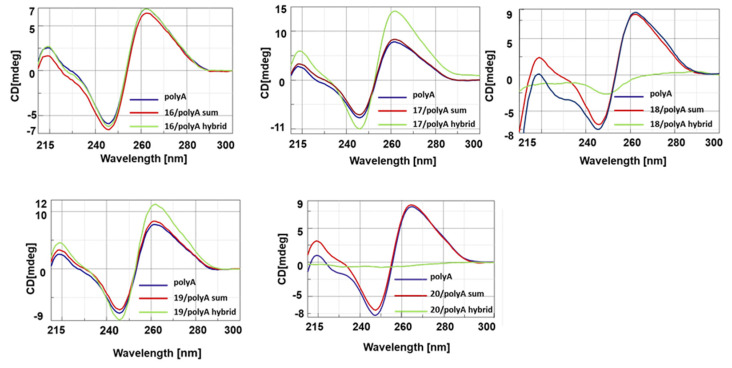
CD spectra of **16**–**20**/PolyA hybrids.

**Figure 3:**
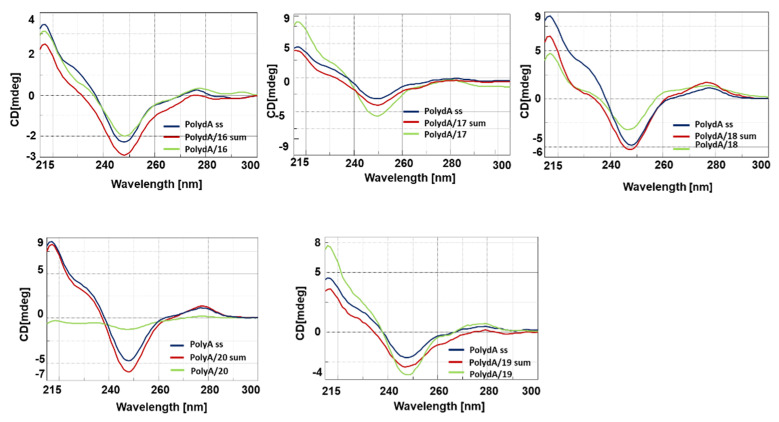
CD spectra of **16**–**20**/PolydA hybrids.

Next, we investigated the spectroscopic behavior of the hybrids composed of nucleopeptides **16**–**20** with polyA and polydA (1:1 ratio in nucleobases), which were selected to reproduce the actual cellular target in vitro. A first CD study was carried out on the library of nucleopeptides in which *L*-Arg residues (**20**) were replaced with *L*-Asp (**16**) or *L*-Glu (**17**) or *L*-Lys (**18**) or *L*-His (**19**). The CD spectra of **16**–**20**/polyA (Figure 2) and **16**–**20**/polydA (Figure 3) hybrids exhibited hypochromic or hyperchromic effects depending on the cationic or anionic character of the side chain of underivatized amino acids. Indeed, except for **19**, cationic nucleopeptides **18** and **20** formed hybrids both polydA and polyA characterized by intense hypochromicity in CD spectra. Conversely, anionic nucleopeptides **16** and **17** produced hybrids with a different spectroscopic behavior; in particular, if compared with the nucleic acid ss counterparts, **17**/polydA and mainly **17**/polyA hybrids showed hyperchromic CD profiles while the dichroic bands of the spectra of **16**/polydA and **16**/polyA appeared slightly increased and reduced, respectively. Based on the specific hallmarks of the CD profiles, which appeared more evident for polyA hybrids with respect to polydA hybrids, we were able to formulate a correlation between the pKa3 values of nucleopeptides **16**–**20** and the spectroscopic behaviors of their resulting hybrids. Indeed, a common hypochromic effect was observed for nucleopeptides **18** (lysine pKa3 = 10.53) and **20** (arginine pKa3 = 12.48), that predominantly exist in their cationic state at pH = 7. Interestingly, the conformation of **18** and **20**/nucleic acid hybrids seemed to resemble the nucleic acid condensation effects described for the histone-like peptides, which are notoriously rich in basic residues, including lysine and arginine [27]. However, RNA condensation results were less wrapped than DNA due to the higher rigidity and sensitivity of the RNA molecule. On the contrary, both anionic nucleopeptides **16** and **17**, having a pKa3 of 3.65 (aspartic acid) and 4.25 (glutamic acid) respectively, formed hybrids characterized by hyperchromic CD spectra. This result led us to hypothesize that the presence of negatively charged side chains in the nucleopeptides does not prevent the formation of hybrids with polyA at pH and ionic strength close to the physiological ones. This hypothesis is supported by the literature for different anionic modified PNAs [28,29,30]. In line with this clustering, the His containing nucleopeptide (**19**) behaved mostly as a non-cationic nucleopeptide, because at pH = 7.00, the percentage of protonated imidazole side chains (histidine pKa3 = 6.00) was not sufficient to produce hypochromic effects.

The thermal stability (melting temperature, Tm) of hybrids involving nucleopeptides **16**–**20**, both polyA and polydA complements, was determined by CD experiments and compared with Tm of the hexathymine oligodeoxynucleotide (T6)/PolyA and PolydA (Table 1). CD melting experiments indicated **20** as the nucleopeptide that formed the most thermal stable hybrids with both polyA and polydA. These results encouraged us to synthesize and study the RNA and DNA binding properties of a new set of congeners of **20**, namely nucleopeptides **31**–**33**, having as an underivatized cationic residue *L*-Arg, and *L*-Dap, *L*-Dab, *L*-Orn, as thymine-functionalized amino acids, respectively.

**Figure 4:**
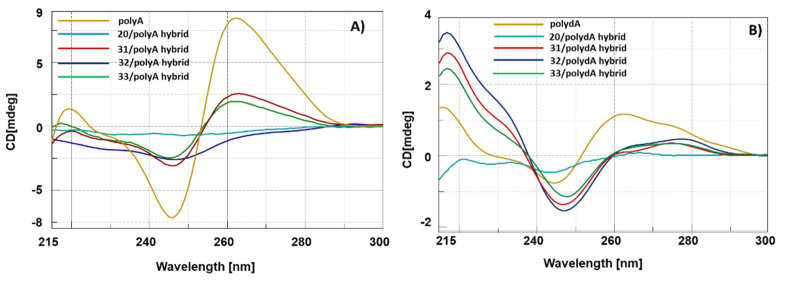
CD spectra of **31**–**33** and **20**/PolyA (**A**) and PolydA (**B**) hybrids.

The ensuing CD studies were carried out on the hybrids composed of nucleopeptides **31**–**33** with PolyA and PolydA to correlate the potential conformational differences with the number of methylene groups spacing the nucleobases from the backbone. As depicted in Figure 4A and 4B, nucleopeptides **31**–**33** formed hybrids with both the nucleic acid counterparts, providing CD spectra that are clearly different from PolyA and PolydA single strands. In the case of **31**–**33**/PolyA hybrids (Figure 4A), the spectra exhibited strong hypochromic variations of diagnostic bands at 218, 247 and 267 nm in respect to the PolyA ss. Interestingly, we rationalized a correlation between the hypochromic effect and the length of the nucleobase linker, which was less intense for nucleopeptides containing an odd number of methylene groups in the nucleoamino acid side chains. Analogously, CD spectra obtained from the hybridization of polydA with nucleopeptides **31** and **33** showed less intense bands (Figure 4B). Surprisingly, hybridization of **32** with polydA resulted in the enhancement of ellipticity of the positive band and in the shift of the CD signals. In particular, both positive bands at 278 nm and the negative band at 247 nm of PolydA shifted at 264 and 244 nm, respectively, suggesting that **32** induced unique conformational changes in the resulting hybrid.

The thermal stability of hybrids involving nucleopeptides **31**–**33** and polyA and polydA complements was determined by CD and UV experiments and compared with the Tm of the (T6)/polyA and **20**/polyA hybrids (Table 2).

It is worth noting that the highest thermal stability was observed for **33**/polyA hybrid (Tm = 60 °C), revealing that **33** could represent the best RNA binder even if compared to arginine containing nucleopeptides **20** and **32** (Tm = 45 °C and 54 °C, respectively). On the other hand, **32** turned out as the best DNA binder, providing the highest Tm value (Tm = 59 °C) among polydA hybrids. Finally, **31** turned out to be the less efficient polyA and polydA binder, providing the lowest Tm value among hybrids.

**Figure 5:**
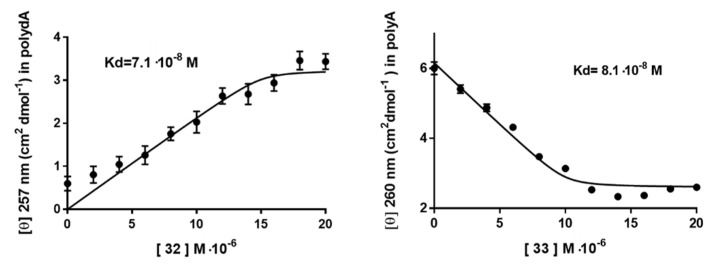
CD titration data of polydA and polyA, with **32** and **33**, respectively.

In order to highlight differences in terms of binding properties with polyA and polydA, CD titration experiments were also performed with **32** and **33** with increasing concentrations of nucleopeptides in the presence of a constant concentration of nucleic acid target. Briefly, the titration experiments were performed by adding increasing concentrations of the nucleopeptides **32** or **33** (0, 1.87, 3.76, 5.60, 7.47, 9.33, 11.20, 13.07, 14.93, 16.8, 18.67, 10 μM) to the solution containing the polyA or polydA (10 μM each base) in a solution of 10 mM of phosphate buffer, 100 mM of NaCl at pH 7.0. The resulting small increase in final volume was considered negligible. The CD spectra were acquired at 20 °C, monitoring the changes of the signal at λ = 257 nm for polydA and 260 nm for polyA, respectively. Titrations were continued until no signal change was observed in the CD spectrum, indicating that the saturation point of the polydA or polyA had been reached. The stoichiometry of the complexes was around ~1:1 for **33**/polyA and ~1:1.5 for **32**/polydA, as depicted in Figure 5. This latter result, together with the CD spectrum profile of **32**/polydA hybrid, indicated peculiar features for this complex. The detected changes in the CD intensity were inserted to generate the isothermal curves. Assuming the formation of a 1: 1 complex for each nucleobase also for the hybrid **32**/polydA, we calculated indicative apparent Kd values of the best polydA and polyA ligands **32** and **33**, as shown in Figure 5.

The indicative apparent Kd values for **32** was 7.1·10^−8^ M with polydA and Kd = 6.7·10^−7^ M with polyA (data not shown), whereas for **33** we found Kd values of 8.1·10^−8^ M and 4.9·10^−6^ M with polyA and polydA, (data not shown) respectively. These data confirmed that cationic nucleopeptides **32** and **33** are endowed with higher affinity for polydA and polyA, respectively. As a result, by solely modulating the distance of the nucleobases from the backbone we could observe significant differences in terms of binding selectivity and stability, which are most evident for homologous nucleopeptides **32** and **33** towards DNA and RNA.

### 2.3. Cell Penetration Study

**Figure 6:**
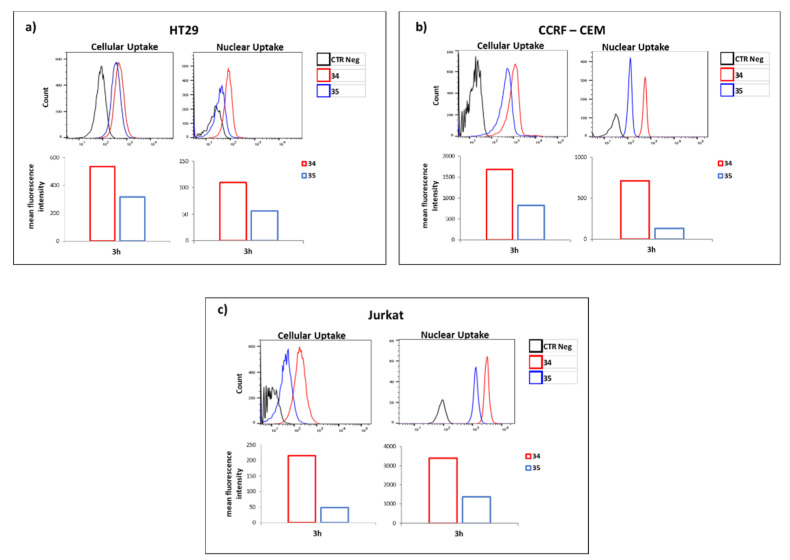
Mean fluorescence intensity of the HT29 (**a**), CCRF-CEM (**b**) and Jurkat (**c**) cells containing **34** and **35** by FACS analysis (lower panel) and the representative results of FACS analysis (upper panel). CTR Neg was the fluorescence of untreated cells.

The promising results achieved with **32** and **33** in terms of DNA and RNA recognition and binding studies prompted us to investigate their capability to cross the cellular membrane and to distribute between cytoplasm and nucleus. Specifically, cell penetration experiments were carried out employing **34** and **35** (the FITC-labelled derivatives of **32** and **33**, respectively) on three different cancer cell lines (Jurkat, HT29 and CEM). As shown in Figure 6, although **34** and **35** are both endowed with the same number of *L*-Arg and differ for the sole nucleobase bearing amino acids (*L*-Dab and *L*-Orn for **34** and **35**, respectively), significant differences of cellular and nucleus permeability were noticed. Indeed, the concentration of **34** in both cytoplasm and nucleus were significantly higher than **35**, suggesting that in addition to the positively charged amino acids, the nucleobase bearing residues also contribute to the uptake process. We reasoned that the observed results could not be solely ascribed to differences of lipophilicity (**34** and **35** differ for a total of six methylene groups), but rather it could be due to the role of the nucleobase functionalized amino acids on the overall nucleopeptide conformations and/or on the DNA/RNA binding. The latter could also account for the higher concentration of **34** at the nuclear area compared to **35**, probably as consequence of its capability to more efficiently interact with cellular DNA compared to **35** (Figure 5). The distribution of **34** in nucleus and cytoplasm confers to this new chemotype the functional features of both CPPs and Nuclear Localization Sequences (NLS), although from a structural point of view **34** did not comply with the canonical requisites previously reported for these two families of peptides [31,32,33].

## 3. Materials and Methods

### 3.1. Materials and Instruments

Nucleopeptides synthesis was performed by using the ultrasonic bath SONOREX RK 52 H, BANDELIN electronic (Berlin, Germany) which had a of volume 1.2 L and inside dimensions 150 × 140 × 100 mm, equipped with a timer control for 1–15 min and continuous (∞) and a heating control (30–80 °C thermostatically adjustable). Ultrasonic frequency was 35 kHz, the ultrasonic nominal output was 60 W, the ultrasonic peak output was 240 W corresponding to 4 times the ultrasonic nominal output. The heating power was 140 W. Usual polypropylene reactors for SPPS were used (ISOLUTE^®®^ SPE filtration column by Biotage, Uppsala, Sweden) supplied with a filter (ISOLUTE^®®^ frits, 20 μm porosity polyethylene frits by Biotage (Uppsala, Sweden), a stopper, and a top cap. The appropriate amount of support was swollen in suitable solvent for 60 min at RT on a Multireax Heidolph, prior to use. For filtering and washing of the resin, a solid-phase extraction (SPE) vacuum manifold (Phenomenex, Torrance, CA, USA) was exploited by using a PTFE universal stopcock that allowed the solvent to pass through the resin and collected in a waste container. Purification of nucleopeptides was carried out by preparative HPLC (Shimadzu HPLC system) equipped with a C18-bounded preparative RP-HPLC column (analytical C18 column Phenomenex Kinetex 21.2 mm × 150 mm, 5 μm) eluting with a gradient (10–90% acetonitrile in water (0.1% TFA) over 20 min; flow rate = 10.0 mL/min; UV detector). Analysis of nucleoeptides oligomers was performed by reversed-phase HPLC on a Shimadzu Prominence HPLC system by using a C18-bounded analytical RP-HPLC column (Phenomenex Kinetex, 4.6 mm × 150 mm, 5 μm) eluted with increasing percentage of ACN in water (from 10 to 90% ACN in water (0.1% TFA) over 20 min; flow rate = 1.0 mL/min; 220 and 260 nm UV detector). Samples were lyophilized for 18 h. Molecular weights of compounds were confirmed by Mass Spectrometry analyses, executed with a matrix assisted laser desorption ionization time-of-flight (MALDI-TOF) micro MX (Waters Co., Manchester, UK) mass spectrometer armed with a pulsed nitrogen laser (k = 337 nm). CD measurements were performed by a JASCO circular dichroism J-810 spectropolarimeter (Mary’s Court, Easton, MD) controlled with a Peltier temperature programmer. An Agilent Cary 300 UV-Vis Spectrophotometer equipped with a temperature controller was employed for UV measurements. HT29 and CCRF-CEM cell lines were obtained from the National Cancer Institute’s Development Therapeutics, Jurkat cells from the American Type Culture Collection (ATCC).

### 3.2. Circular Dicroism Procedures

Stock solutions of oligomers were prepared in sterilized doubly distilled water. Their concentration was assessed measuring by UV at 260 nm using the following molar extinction coefficient: ε 260 (A) = 13.7 mL/(μmole × cm), ε 260 (T) = 8.8 mL/(μmole × cm), at 90 °C. CD spectra and melting curves were acquired on J-810 spectropolarimeter. Both UV and CD spectra were acquired using quartz cells with an optical path length of 1 cm. All hybridization experiments were performed in a 10 mM phosphate buffer, 100 mM NaCl, pH 7.0. Duplexes were prepared by mixing equimolar concentrations of nucleopeptides with polydA (M.W. 330 Da; chain length 250−500 bases) (6 μM each base) and polyA (M.W. 100−500 Da; chain length 2100− bases) (6 μM each base). Annealing was accomplished by incubating the samples at 90 °C for 5 min, then slowly cooling down at RT and finally the samples were kept at 4 °C overnight. CD melting was monitored at λ = 247 nm and at λ = 267 nm for the duplexes with DNA, RNA oligomers, respectively, in the 5−85 °C temperature range, with a scan rate of 1 °C min^−1^. Melting temperatures were measured as the zero point of the second derivative of the melting curves. A 215−300 nm spectral window was applied for CD spectra, keeping the temperature constant at 10 °C. Sum spectra derive from the arithmetic addition of the CD spectra of the individual strands forming the hybrid. The titration experiments were carried out by the addition of increasing the amounts of nucleopeptides **32** and **33** (0, 1.87, 3.76, 5.60, 7.47, 9.33, 11.20, 13.07, 14.93, 16.8, 18.67 μM each base) in a solution containing the single stranded complementary polyA (10 μM each base) and polydA (10 μM each base) in a 10 mM phosphate buffer, 100 mM NaCl, pH 7.0. CD spectra were acquired at 10 °C, monitoring the signal decrements at λ = 260 nm for the polyA and increments at λ = 257 nm for polydA. The detected changes in CD intensity were plotted to generate saturation isotherm curves as a function of added nucleopeptide amounts. Supposing the formation of a 1:1 complex for each nucleobase, we estimated the apparent Kd values by using the following equation:

Q = Qmin − [(Qmin − Qmax)/(2cA)] × {Kd + cT + cA − [(Kd + cT + cA)2 − 4cAcT]1/2},

where Q is the CD intensity of the characteristic hybrid wavelength, Qmin and Qmax are the minimum and maximum values of CD intensity, respectively, and cA and cT are the Adenine and Thymine nucleobase concentrations, respectively (R2 values were always equal to or greater than R2 ≥ 0.90).

### 3.3. Synthesis of Nucleopeptides **16**–**20**, **31**–**33**

A Rink Amide AM-PS resin (55 mg, 0.03 mmol, 0.55 mmol/g) was swelled in DCM/DMF 1:1 (*v*:*v*) over 60 min and then washed with DCM (3 × 1 min) and DMF (3 × 1 min). First, the resin-bound Fmoc protecting group was removed by treating with 20% (*v*/*v*) piperidine solution in DMF (1 × 0.5 min; 1 × 1 min) under ultrasonic irradiation. The coupling reaction was achieved by adding the corresponding Nα-Fmoc amino acid (3 eq) and coupling reagents HBTU/HOBt (3 equiv) suspended in DMF (0.13 M), DIEA (6 equiv), ultrasonic irradiation (10 min). Completion of these steps were qualitatively monitored by Kaiser ninhydrine and TNBS test and quantitatively ascertained by Fmoc UV spectroscopic measurements. After the last Fmoc removal, N-terminal was acetylated by treatment with a mixture of Ac_2_O (5.7 μL, 2 equiv) and DIPEA (15.7 μL, 3 equiv) in DMF (1 mL) over 3 min, ultrasonic irradiation. The Alloc groups were removed by treating the supported full-protected peptide twice with a solution of Tetrakis(triphenylphosphine)palladium (0) (11 mg, 0.01 mmol, 10% mol relative to each Alloc group) and DMBA (123 mg, 0.079 mmol, 4 equiv. relative to each Alloc protective group) in dry DMF/DCM (1:1) for 60 min. The resulting solution was drained off and the resin was washed (3 × 1 min) with DMF and DCM. The catalyst traces were washed out by treating the solid support twice with a DMF solution of potassium N,N-diethylcarbamodithioate (0.06 M, 1.5 mL) for 30 min, under mechanical stirring. To obtain the thyminyl-functionalized nucleopeptides, Thymin-1-yl-acetic acid (108.1 mg, 0.59 mmol, 3 equivalents for each amine group), PyAOP (308 mg, 0.59 mmol), DIPEA (205 μL, 1.08 mmol, 6 equivalents for each amine group) were dissolved in DMF (2 mL) and then added to resin for reacting over 12 h at room temperature.

### 3.4. Synthesis of Nucleopeptides **34**–**35**

Nucleopeptides **34**–**35** were synthesized following the procedures described above. After Fmoc-deprotection at N-terminus of Alloc-protected peptides **22**–**24**, Fmoc-Ahx-COOH spacer (3 eq) was added by using the coupling reagents HBTU/HOBt (3 equiv) suspended in DMF (0.13 M), DIEA (6 equiv), ultrasonic irradiation (10 min). Then, the Alloc groups were removed by treating the supported full-protected peptide twice with a solution of Tetrakis(triphenylphosphine)palladium (0) (0.01 mmol, 10% mol relative to each Alloc group) and DMBA (0. 79 mmol, 4 equivalents relative to each Alloc protective group) in dry DMF/DCM (1:1) for 60 min, RT. The resulting solution was drained off and the resin was washed (3 × 1 min) with DMF and DCM. The catalyst traces were washed out by treating the support twice with a DMF solution of potassium N,N-diethylcarbamodithioate (0.06 M, 1.5 mL) for 30 min under mechanical stirring. To obtain the thyminyl-functionalized nucleopeptides, Thymin-1-yl-acetic acid and PyAOP (3 equivalents for each amine group) with DIPEA (6 equivalents for each amine group) were dissolved in DMF (2 mL) and then added to resin for reacting over 12 h at room temperature. The nascent nucleopeptides were removed from the Fmoc protective group as described above and then labeled on a terminal primary amine by reacting with fluorescein-5- isothiocyanate (FITC) (6 equivalents) and DIEA (12 equivalents) in DMF (2 mL); the reaction mixture was shaken for 12 h in the dark. The unreacted free amines were acylated with a solution of Ac_2_O (2 eq. relative to each free amine) and DIPEA (3 equivalents) in DMF (1 mL) over 10 min.

### 3.5. Cleavage, Purification and Characterization

All the resulting resin-bound nucleopeptides **16**–**20**, **31**–**35** were carefully washed with DMF (5 × 1 min), DCM (5 × 1 min), and Et_2_O (2 × 1 min) and then dried. Eventually, they were cleaved from the resin by treatment with TFA/TIS/H_2_O (95:2.5:2.5, 3 mL) for 3 h at RT (in the dark for labeled-nucleopeptides **34** and **35**). After removal of the resin by filtration, the crude nucleopeptides were precipitated from the TFA solution, diluting to 15 mL with cold ethylic ether, and then centrifuged (6000 rpm × 15 min). After, the supernatant was removed, the precipitate was washed with Et_2_O. The obtained wet solid was dried overnight under reduced pressure, dissolved in water/acetonitrile (9:1) and purified by reverse-phase HPLC (solvent A: water + 0.1% TFA; solvent B: acetonitrile + 0.1% TFA; from 0 to 90% of solvent B over 25 min, flow rate: 10 mL min^−1^). Fractions of interest were dried under reduced pressure to remove the organic solvent and then lyophilized (in the dark for **34** and **35**). Obtained nucleopeptides were characterized by analytical HPLC and MALDI-TOF spectrometry. Yield, purity, retention times, and analytical data are reported in Table 3.

### 3.6. Cellular and Nuclear Uptake Procedures

The fluorescence of Fluorescein Isothiocyanate (FITC) (excitation 488 nm, emission 525 nm) was used to evaluate the intracellular and intranuclear nucleopeptides uptake using flow cytometry. HT29, human colon cancer cells, CCRF-CEM and Jurkat, leukemic T cells, were grown in RPMI-1640 with 10% FBS and 2 mM glutamine in a humidified incubator, containing 5% CO2 at 37 °C. Cells (1 × 10^6^) were treated with FITC-labelled-34 and 35 (10 µM) for 3 h at 37 °C. After 3 h the cells were rinsed twice with PBS1. To evaluate cellular uptake, the cell pellets were suspended in 500 µL of PBS and analyzed by flow cytometry. For nuclear uptake, the nuclei were extracted with PBS-Triton (0.5%) for 15 min at room temperature (RT), washed with PBS-Triton (0.1%), suspended in 500 µL of PBS and analyzed by FACS ARIA III flow cytometry (BD Bioscience, Mountain View, CA, USA) with DIVA software 8.01. A minimum of 20,000 events for each sample were collected. The uptake was evaluated as mean fluorescence and was analyzed using FlowJo 10.7.1 software.

## 4. Conclusions

In this paper, we describe the efficient synthesis of two sets of homo-thymine nucleopeptides with the aim of investigating the role of the underivatized and of the nucleobase bearing amino acids on the recognition and binding events with complementary polyA and polydA oligonucleotides. We found that the nature of the underivatized amino acids (both anionic and cationic) affects both the binding and thermal stability of DNA/RNA oligonucleotide-nucleopeptide hybrids.

On the other hand, by focusing on the nucleobase-bearing amino acids (NBAs), we observed a correlation between the side chain length and the conformational behavior. Nucleopeptides where nucleobases were outdistanced from the peptide backbone by an odd or an even number of methylene groups showed similar conformational behavior, respectively. Furthermore, those harnessing of *L*-Dab (32) and *L*-Orn (33) as NBAs differed for the strength of their interaction with polydA and polyA counterparts, respectively.

In addition, considering the membrane penetration potential of the polycationic macromolecules (CPP) we found different cellular and nuclear localization between Arg-based nucleopeptides having the same number of positive charges but a different NBAs side chain length. This evidence suggests that the nucleobase-bearing amino acids play a pivotal role in defining the elective oligonucleotide binding partner and the cell/nuclear uptake properties of nucleopeptides. Finally, the ability of these cationic nucleopeptides to reach the nucleus and to bind DNA raises new opportunities, for example, in the design of distinctive nuclear DNA interacting carriers able to deliver DNA damage agents, directly and selectively to the target.

## Figures and Tables

**Table 1:** Tm values of **16**–**20** with their respective nucleic acid targets.

Entry	Sequence	PolydA (Tm)	PolyA (Tm)
**T_6_**	3′-TTTTTT-5′	25	35
**16**	Ac-Lys(T)-Asp-Lys(T)-Asp-Lys(T)-Asp-Lys(T)-Asp-Lys(T)-Asp-Lys(T)-Asp-CONH_2_	35	42
**17**	Ac-Lys(T)-Glu-Lys(T)-Glu-Lys(T)-Glu-Lys(T)-Glu-Lys(T)-Glu-Lys(T)-Glu-CONH_2_	36	35
**18**	Ac-Lys(T)-Lys-Lys(T)-Lys-Lys(T)-Lys-Lys(T)-Lys-Lys(T)-Lys-Lys(T)-Lys-CONH_2_	26	35
**19**	Ac-Lys(T)-His-Lys(T)-His-Lys(T)-His-Lys(T)-His-Lys(T)-His-Lys(T)-His-CONH_2_	37	25
**20**	Ac-Lys(T)-Arg-Lys(T)-Arg-Lys(T)-Arg-Lys(T)-Arg-Lys(T)-Arg-Lys(T)-Arg-CONH_2_	36	45

**Table 2:** Tm values of **16**–**20** with their respective nucleic acid targets.

Entry	Sequence	PolydA (Tm)	PolyA (Tm)
**T_6_**	3′-TTTTTT-5′	25	35
**31**	Ac-Dap(T)-Arg-Dap(T)-Arg-Dap(T)-Arg-Dap(T)-Arg-Dap(T)-Arg-Dap(T)-Arg-CONH_2_	<10	**30**
**32**	Ac-Dab(T)-Arg-Dab(T)-Arg-Dab(T)-Arg-Dab(T)-Arg-Dab(T)-Arg-Dab(T)-Arg-CONH_2_	59	54
**33**	Ac-Orn(T)-Arg-Orn(T)-Arg-Orn(T)-Arg-Orn(T)-Arg-Orn(T)-Arg-Orn(T)-Arg-CONH_2_	40	60
**20**	Ac-Lys(T)-Arg-Lys(T)-Arg-Lys(T)-Arg-Lys(T)-Arg-Lys(T)-Arg-Lys(T)-Arg-CONH_2_	36	45

**Table 3:** Yield, purity, retention times, and analytical data of the described nucleopeptides.

Entry	Yield (%)	tR (min)	Purity	Mass Calcd	Mass Found
**16**	56%	9.93	≥ 95%	2515.43	2538.11 * 2513.92 **
**17**	61%	10.32	≥ 95%	2599.58	2622.13 * 2598.01 **
**18**	72%	8.76	≥ 95%	2593.94	2594.17 ***
**19**	68%	9.27	≥ 95%	2647.74	2648.11 ***
**20**	79%	9.29	≥ 95%	2763.04	2762.45 ***
**31**	69%	8.68	≥ 95%	2509.54	2510.14 ***
**32**	71%	9.12	≥ 95%	2509.70	2594.42 ***
**33**	77%	9.47	≥ 95%	2677.86	2678.52 ***
**34**	60%	11.71	≥ 95%	3054.25	3054.71 ***
**35**	63%	9.40	≥ 95%	3136.45	3137.77 ***

* [M + Na]+, ** [M-H]−, *** [M + H]^+^.

## Data Availability

Not applicable.
